# Real-world multidisciplinary outcomes of onasemnogene abeparvovec monotherapy in patients with spinal muscular atrophy type 1: experience of the French cohort in the first three years of treatment

**DOI:** 10.1186/s13023-024-03326-3

**Published:** 2024-09-13

**Authors:** Isabelle Desguerre, Rémi Barrois, Frédérique Audic, Christine Barnerias, Brigitte Chabrol, Jean Baptiste Davion, Julien Durigneux, Caroline Espil-Taris, Marta Gomez-Garcia de la Banda, Marine Guichard, Arnaud Isapof, Marie Christine Nougues, Vincent Laugel, Laure Le Goff, Sandra Mercier, Anne Pervillé, Christian Richelme, Marie Thibaud, Catherine Sarret, Cyril Schweitzer, Hervé Testard, Valérie Trommsdorff, Catherine Vanhulle, Ulrike Walther-Louvier, Cécilia Altuzarra, Mondher Chouchane, Juliette Ropars, Susana Quijano-Roy, Claude Cances

**Affiliations:** 1 IHU Imagine, Paris University, 24, Boulevard du Montparnasse, 75015 Paris, France; 2 Department of Pediatric Neurology, French Reference Center for Neuromuscular Diseases, AP-HP, Hôpital Necker-Enfants Malades, 149 rue de sèvres, 75015 Paris, France; 3 Department of Pediatric Neurology, French Reference Center for Neuromuscular Diseases, Hôpital Timone Enfants, 264 rue Saint-Pierre, 13385 Marseille, France; 4 Department of Pediatric Neurology, French Reference Center for Neuromuscular Diseases, Lille University Hospital Center, 2 avenue Oscar Lambret, 59000 Lille, France; 5 Department of Pediatric Neurology, French Reference Center for Neuromuscular Diseases, Angers University Hospital Center, 4 rue Larrey, 49933 Angers, France; 6 Department of Pediatric Neurology, French Reference Center for Neuromuscular Diseases, Pellegrin University Hospital Center, Hôpital des Enfants, place Amélie-Raba-Léon, 33086 Bordeaux, France; 7 Pediatric Neurology and ICU Department, Garches Reference Center for Neuromuscular Diseases (NEIF for FILNEMUS; RPC for Euro-NMD ERN), AP-HP Paris-Saclay Université, Hôpital Raymond Poincaré (UVSQ), 104 boulevard Raymond Poincaré, 92380 Garches, France; 8 Department of Pediatric Neurology and Handicaps, French Competence Center for Neuromuscular Diseases, Boulevard Tonnellé, Hôpital Clocheville, 2 Boulevard Tonnellé, 37000 Tours, France; 9 Department of Pediatric Neurology, AP-HP, French Reference Center for Neuromuscular Diseases, Hôpital Armand Trousseau, 26 avenue du Docteur Arnold-Netter, 75012 Paris, France; 10 Department of Pediatric Neurology, French Reference Center for Neuromuscular Diseases, Strasbourg University Hospital Center, Hôpital de Hautepierre, 1 avenue Molière, 67098 Strasbourg, France; 11 Department of Neuromuscular Pathology, French Reference Center for Neuromuscular Diseases, Hôpital Femme Mère Enfant, Hospices Civils de Lyon, -Bron, 59 boulevard Pinel, 69677 Lyon-Bron, France; 12 Department of Medical Genetics, French Reference Center for Neuromuscular Diseases, Nantes University Hospital Center, 1 Place Alexis-Ricordeau, 44093 Nantes, France; 13 Department of Pediatrics, French Competence Center for Neuromuscular Diseases, Hôpital d’Enfants ASFA, CS 81010, 97404 Saint Denis Cedex, Réunion France; 14 Department of Pediatric Neurology, French Reference Center for Neuromuscular Diseases, Nice University Hospital Center, Hôpital Lenval, 57 Avenue de la Californie, 06200 Nice, France; 15 Department of Pediatrics, French Reference Center for Neuromuscular Diseases, American Memorial Hospital, Reims University Hospital Center, 49 Rue Cognacq Jay, 51092 Reims, France; 16 CMR Neuromusculaire, French Reference Center for Neuromuscular Diseases, Clermont-Ferrand University Hospital Center, Clermont-Ferrand, France; 17 Department of Infant Medicine, French Reference Center for Neuromuscular Diseases, Nancy University Hospital Center, Rue du Morvan, 54511 Vandoeuvre lès Nancy, France; 18 Department of Pediatric Neurology, French Competence Center for Neuromuscular Diseases, Grenoble University Hospital Center, Hôpital Couple Enfant, Quai Yermolof, 38700 Grenoble, France; 19 Department of Pediatrics, French Reference Center for Neuromuscular Diseases, University Hospital Center, Avenue François Mitterrand, BP 350, 97448 Saint Pierre Cedex, Réunion France; 20 Department of Pediatrics, French Competence Center for Neuromuscular Diseases, Rouen University Hospital Center, Charles Nicolle, 1 Rue de Germont, 76031 Rouen, France; 21 Department of Pediatric Neurology, French Greater South‒West Reference Center for Neuromuscular Diseases, Hôpital Gui de Chauliac, University Hospital Center Montpellier, 80 Avenue Augustin Fliche, 34295 Montpellier, France; 22 Department of Pediatrics, French Reference Center for Neuromuscular Diseases, Besançon University Hospital Center - Hôpital Jean Minjoz, 3 boulevard A. Fleming, 25030 Besançon, France; 23 Department of Pediatric Neurology, French Competence Center for Neuromuscular Diseases, Dijon University Hospital Center, Hôpital d’Enfants, 14 rue Paul Gaffarel, 21079 Dijon, France; 24 LaTIM INSERM UMR 1101, French Reference Center for Neuromuscular Diseases Brest University Hospital Center, Hôpital Morvan, Boulevard Tanguy Prigent, 29609 Brest, France; 25 Department of Pediatric Neurology, French Greater South‒West Reference Center for Neuromuscular Diseases, Hôpital des Enfants, University Hospital Center Toulouse, 330 av de Grande Bretagne-TSA, 31059 Toulouse, France

**Keywords:** Spinal muscular atrophy, Gene transfer therapy, Real-world outcome

## Abstract

**Background:**

Spinal muscular atrophy type 1 (SMA1) is the most severe and early form of SMA, a genetic disease with motor neuron degeneration. Onasemnogene abeparvovec gene transfer therapy (GT) has changed the natural history of SMA1, but real-world data are scarce.

**Methods:**

A French national expert committee identified 95 newly diagnosed treatment-naive SMA1 patients between June 2019 and June 2022. We prospectively report on children treated with GT as the first and only therapy who had more than one-year of follow-up.

**Results:**

Forty-six SMA1 patients received GT. Twelve patients received other treatments. Patients with respiratory insufficiency were oriented toward palliative care after discussion with families. Twenty-nine of the treated patients with more than 12 months of follow-up were included in the follow-up analysis. Among them, 17 had 24 months of follow-up. The mean age at treatment was 7.5 (2.1–12.5) months. Twenty-two patients had two *SMN2* copies, and seven had three copies. One infant died in the month following GT due to severe thrombotic microangiopathy, and another died due to respiratory distress. Among the 17 patients with 24 months of follow-up, 90% required spinal bracing (15/17), three patients required nocturnal noninvasive ventilation, and two needed gastrostomy. Concerning motor milestones at the 24-month follow-up, all patients held their head, 15/17 sat for 30 s unassisted, and 12/17 stood with aid. Motor scores (CHOPINTEND and HINE-2) and thoracic circumference significantly improved in all patients.

**Conclusions:**

Our study shows favorable motor outcomes and preserved respiratory and feeding functions in treatment-naive SMA1 infants treated by GT as the first and only therapy before respiratory and bulbar dysfunctions occurred. Nevertheless, almost all patients developed spinal deformities.

## Introduction

Spinal muscular atrophy (SMA) is an autosomal, recessive, neuromuscular disease with an incidence of approximately 1:10,000 live births [1]. SMA is characterized by progressive motor neuron degeneration leading to proximal muscle weakness and atrophy.

Without disease-modifying treatments, SMA phenotypes are divided into five types according to the age of onset and maximal motor acquisition, from type 0 (symptoms at birth) to type 4 (adult onset). In SMA type 1 (SMA1), symptoms occur in the first 6 months of life, and three subtypes are distinguished (1a, 1b and 1c). In SMA types 1a and 1b, head control is never achieved and signs appear within 4 weeks of life (1a) or within the first 3 months (1b). In SMA type 1c patients, head support is achieved for a while and the onset of symptoms occurs between 3 and 6 months [2, 3]. SMA1 is rapidly progressive, and in the absence of continuous vital support, infants die within the first 2 years of life of respiratory insufficiency [4]. SMA severity is modulated by the number of copies of *SMN2*, a second “protector” gene [5], and SMA1 infants often have two *SMN2* copies and may even have three.

Recently, new therapies have changed the prognosis of SMA patients. Three treatments have been approved by the US Food and Drug Administration (FDA) and the European Medicines Agency (EMA). Nusinersen: Spinraza^®^ and risdiplam: Évrysdi^®^ (*SMN2* pre-mRNA splicing modifiers) were proposed in France with an early access program, respectively in 2017 and 2020. Onasemnogene abeparvovec, commercialized as Zolgensma^®^ [a gene replacement therapy that offsets the defective *SMN1* gene with a therapeutic functional *SMN1* copy delivered by adeno-associated virus 9 (AVV9)] was approved in France in April 2019.

Recent papers (from open-label, phase I and III trials and from real-world experience) compiled in Blair 2022 showed that onasemnogene abeparvovec prolonged event-free survival and improved motor function and motor milestone achievements [6]. Nevertheless, regarding the efficacy and medium- to long-term motor outcomes of these treatments, few real-world data have been published [7–12]. The main drawbacks of the existing studies are the small numbers of reported subjects [10, 11], the multiplicity of treatments [12], and the heterogeneity of the population analyzed with regard to age, SMA type and *SMN2* copy number [7]. Furthermore, although clinical score improvement after GT is important, patient-oriented outcomes such as motor milestone achievements and the need for ventilation or nutritional support should also be emphasized [7].

Onasemnogene abeparvovec is generally well-tolerated, although transitory liver enzyme increases or thrombocytopenia have been documented [8, 13–15]. Moreover, severe adverse events such as hepatitis and thrombotic microangiopathy (TMA) have been described during the first 2 weeks after infusion [16–19]. Rare deaths have been reported to be induced by TMA or hepatocellular failure [16, 20, 21]. One case of epithelioid neoplasm of the spinal cord after GT was also recently reported [22].

Since 2011, the French neuromuscular network, FILNEMUS (from French: FILières des maladies NEuroMUSculaires), has included 23 reference centers (htpp://filnemus.fr). Treatment indications in France are currently restricted to SMA1 and SMA2 children under 2 years old and weighing less than 12 kg [23]. As the clinical benefits of treatment in severely ill infants with bulbar impairment and/or respiratory insufficiency are unclear given the absence of data, the FILNEMUS national expert committee restricts GT for SMA1 infants to those without significant respiratory or bulbar impairment.

We report original real-world data and major adverse events from naive SMA1 patients treated by onasemnogene abeparvovec as a monotherapy in France between June 2019 and June 2022, all with 12 to 24 months of follow-up.

## Methods

### Patients

Between June 2019 and June 2022, all consecutive SMA1 patients diagnosed in one of the 23 French pediatric reference centers for neuromuscular disorders (with genetically confirmed diagnosis of homozygous *SMN1* exon 7 deletions and the number of *SMN2* copies) were identified by the FILNEMUS national expert committee through bimonthly virtual meetings (or ad hoc urgent supplementary meetings). This national commission was established in France in 2017 at the request of the reference center clinicians to ensure a group of expert clinicians able to define the indications for innovative SMA therapies in real life. Concerning onasemnogene abeparovec (GT), the commission’s validation of an indication is mandatory in France to obtain the drug, which can be administered only in expert centers authorized by the health authorities for gene transfer therapy prescription and delivery.

### Inclusion/exclusion criteria

The various therapeutic options (nusinersen since 2017, onasemnogene abeparvovec since 2019, and risdiplam since 2020) were discussed for each patient by the expert committee.

All patients treated by GT as the first and only line with more than 12 months follow-up after treatment were included in this study. Inclusion criteria were:

- genetically confirmed diagnosis of SMA
- clinical phenotype of SMA type 1
- more than 12 months follow-up at the end of the study period.

Exclusion criteria were:

- clinical phenotype of SMA other than type 1
- severe respiratory impairment or bulbar signs (*i.e.,* need for ventilatory support or continuous enteral feeding; a TC/HC ratio < 0.85) or a profound motor deficit (evaluated by HINE-2, CHOPINTEND and clinical examination). In this case, palliative care was recommended.
- recent vaccination, logistic difficulties, or a positive PCR result for a respiratory virus (COVID, SRV, etc.). In the latter case, nusinersen or risdiplam was proposed as the first-line treatment until the viral PCR was negativized, allowing the patient to switch to GT as a second line.
- less than 12 months follow-up after treatment at the end of the study period.

The parents were informed of the different treatment possibilities and the need for the expert committee’s approval. The final decision of the committee was made with the child’s best interests in mind, most often with the parents’ full agreement [24].

### GT infusion procedure

After confirming AAV9 antibody negativity, the onasemnogene abeparvovec was administered in the intensive care unit of one of the accredited French hospitals (currently 14) through a central venous catheter after thorough clinical and biological evaluation: viral investigations (nasal PCR for SARS-CoV-2 and multiplex viral PCR) and respiratory and cardiac evaluations (ECG and echocardiography). Patients stayed at the hospital at least 3 days after the infusion and for up to a week.

Before summer 2021, prednisolone (1 mg/kg/day) was administered for 1 month (increased if required in the case of hypertransaminemia or thrombocytopenia) and was slowly and carefully decreased over 1 month or more as transaminase and thrombocytosis levels normalized. After the fatal outcome in a child due to TMA in January 2021, a reinforced protocol with an increased initial dose (2 mg/kg/day) was proposed and variably maintained during the first 2 weeks, depending on blood test results and clinical course, then reduced to 1 mg/kg/day for 1 month, and slowly tapered in the following weeks, depending on blood results and clinical course.

Vaccinations, including palivizumab, were given in accordance with the French vaccine program to prevent severe SRV infections during the first two winters.

### Data collection and analyses

This prospective study is registered under approval statement number MR004: 2206723 v 0. Genetic results (*SMN1* deletion and *SMN2* copy number), age at onset and subtype of SMA (type 1a, 1b, 1c or presymptomatic) were collected at baseline. Given that the included patients were treatment-naive and already in the motor regression phase of the disease at diagnosis, the types of SMA (age at symptom onset and highest developmental keystone reached) were applicable for their initial description, as no motor milestone achievement was expected in the natural course of the disease without treatment.

Developmental milestones (head support, unaided sitting and standing a few seconds with aid), need for respiratory or feeding support (NIV, gastrostomy), spinal deformity and need for a spinal brace, motor function scores (HINE-2 and CHOPINTEND) and TC/HC ratio (a simple measure reflecting pulmonary growth associated with mortality in SMA natural history [25, 26]) were collected at baseline and 0, 1, 3, 6, 12, 18 and 24 months (M) after GT. Unaided sitting was defined as the ability to sit for 30 s without aid. In this study, NIV patients refers to patients with NIV during sleeping times. No patient required NIV for longer periods. All patients who needed nutritional support underwent gastrostomy. No patient had a gastric tube or modified diet. In the study, nutritional support refers to gastrostomy.

Classical biological assessments to monitor tolerance of GT (liver enzymes, liver function, complete blood count) were regularly performed in accordance with the recommendations. Only severe adverse events are reported here.

### Statistical analysis

All the statistical analyses were performed with Excel and R software (version 4.0.2). Means were compared using Student’s t test (Excel T.TEST function). The p values are presented for descriptive purposes only. A p value less than 0.05 indicated statistical significance.

The onasemnogene abeparvovec treatment procedure and follow-up were systematically reported to the French SMA Registry (R-SMA).

## Results

### Flow chart

**Fig. 1:**
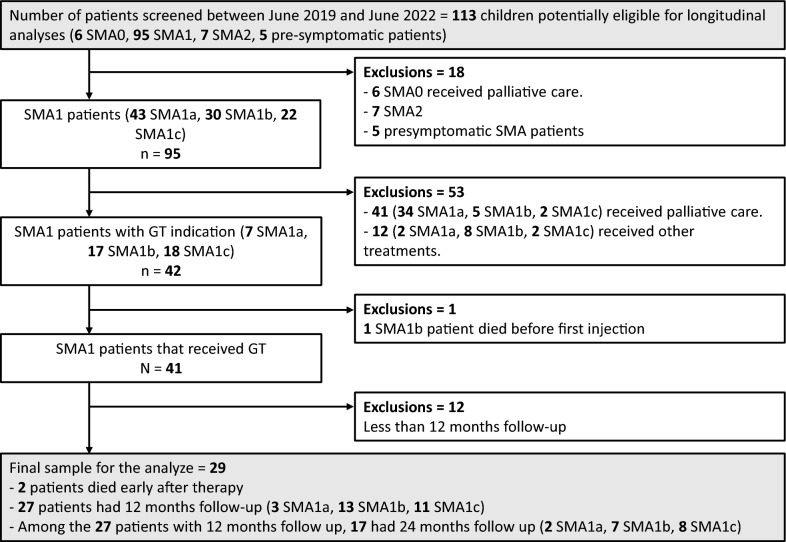
Flow chart detailing SMA patients diagnosed between June 2019 and June 2022 who were eligible for gene transfer therapy. Non-SMA1 patients were excluded from this study, and all SMA1 patients were evaluated by discussion with the FILNEMUS national expert committee. The different treatment options for SMA1 patients discussed by the expert committee are shown, and SMA1 patients with more than 12 months of follow-up were included in the follow-up analysis

Between June 2019 and June 2022, 113 patients were diagnosed with SMA and were discussed at the FILNEMUS bimonthly national expert committee meetings. Among the 113 children, six had SMA0, seven had SMA2, and five were presymptomatic (diagnosed because of a previous case in the family); these patients were excluded from the study. All five presymptomatic patients received GT (see flow chart in Fig. 1).

The remaining 95 patients discussed at the committee meetings were SMA1. Among them, 41 were oriented toward palliative care (34 SMA1a = 83%). Of those for whom a treatment was retained, 42 received GT as a first-line drug. Twelve received an *SMN2* modifier (nusinersen or risdiplam) as a first-line drug or as a bridge to a later GT administration; these patients were excluded from the follow-up analysis. Among these 12 patients, nusinersen was proposed to four patients because of clinical issues, particularly a poor “risk–benefit” balance (2 patients with persistent unexplained hepatic cytolysis, 1 patient with persistent respiratory infection and a positive viral PCR, and 1 patient with cardiopathy). Nusinersen or risdiplam was proposed for the eight other patients because of suboptimal agreement with parents on the treatment strategy (GT refusal or compassionate treatment for patients receiving palliative care recommendations). In these patients, drug selection was decided by the local clinician in charge of the patient.

Among the 42 SMA1 infants with SMA1 and GT prescriptions, one died from respiratory failure due to acute respiratory infection in the context of rapid progression of the disease before receiving GT. Forty-one patients with SMA1 thus received GT. Among them, 29 patients with more than 12 months of follow-up were included in the follow-up analysis. One patient died in the month after GT due to fatal TMA, and one due to respiratory infection. Baseline characteristics for these two patients were, respectively: female, 4.2 months at infusion, type 1b, *SMN2* copy number: 2, TC/HC ratio: 1, and CHOPINTEND score: 17; and male, 6.7 months at infusion, type 1b, *SMN2* copy number: 2, TC/HC ratio: 0.98, and CHOPINTEND score: 21.

### Baseline demographic and clinical characteristics

The baseline characteristics of these 29 patients are given in Table 1 (gender, age at treatment infusion, weight, *SMN2* copy number, clinical phenotype, CHOPINTEND score, HINE-2 score, TC/HC ratio). No patient at baseline required nutritional support (gastrostomy, gastric tube feeding or modified diet). One patient had NIV for less than 12 h per day because of recurrent atelectasis. Concerning the number of *SMN2* copies, the seven patients with three copies of *SMN2* at diagnosis had a less severe phenotype, were older (mean age: 10.6 vs. 6.6 months), and had better motor scores (mean CHOPINTEND score: 37.3 vs. 24.1 and mean HINE-2 score: 7 vs. 3) than did the 22 patients with two *SMN2* copies (Table 1).

**Table 1:** Baseline demographic and clinical characteristics of all patients treated with gene replacement therapy included in the follow-up analysis (i.e., with more than 12 months of follow-up in June 2022) [n = 29]

Caracteristics	All patient (N = 29)	2 copies (N = 22)	3 copies (N = 7)
Sex ratio M/F	10/19	8/14	2/5
SMN type			
1a	3	3	0
1b	15	15	0
1c	11	4	7
Mean age (range)—month	7.5 (2.1–12.5)	6.6 (2.1–12.1)	10.6 (6.5–12.5)
Mean height (range)—cm	67.4 (55–78)	65.5 (55–75)	73 (69–78)
Mean weight (range)—kg	7.4 (4.6–9.6)	7.1 (4.6–9.2)	8.5 (7.1–9.6)
TC/HC (range)	0.98 (0.88–1.05)	0.97 ( 0.88–1.05)	1.00 (0.94–1.04)
Mean HINE-2 scale	4 (0–9)	3 (0–7)	7 (4–9)
Mean CHOPINTEND scale	27.3 (9–50)	24.1 (9–37)	37.3 (27–50)
Patient with clinical support—number			
Gastrostomy	0	0	0
NIV	1	1	0
Spinal brace	0	0	0

*NIV* noninvasive ventilation, *SMA* spinal muscular atrophy

### Changes in key motor milestones and vital support after onasemnogene abeparvovec treatment

After 24 months of follow-up (n = 17), all patients reached head holding. All patients with three *SMN2* copies reached 30 s of unaided sitting, whereas 85% (11 patients) of those with two copies reached this level. All patients with three *SMN2* copies could stand for a few seconds with aid, whereas 62% (8 patients) of those with two copies could do so. No patient with three copies of *SMN2* required NIV, whereas 23% (3 patients) of those with two copies did. No patient with three copies of *SMN2* required gastrostomy, whereas 15% (2 patients) of those with two copies of SMN2 required nutritional support. No patient needed a gastric tube or modified diet. Despite motor improvement, 88% of the patients (15/17) required a brace to treat spinal collapse or scoliosis. The brace was prescribed earlier in patients with two *SMN2* copies (at M12) than in patients with three copies (at M24) (Tables 2, 3).

**Table 2:**
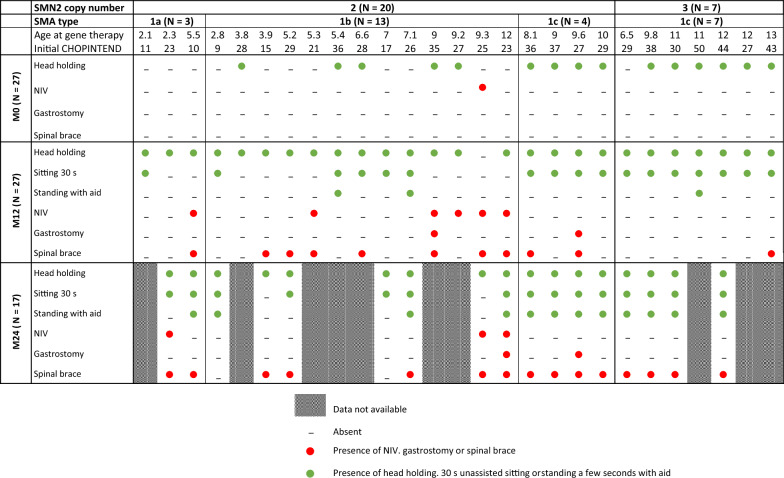
Individual real-world endpoints from baseline to the 24-month follow-up after gene replacement therapy. Patients were first sorted from left to right by SMN2 copy number, second by SMA type, and third by age at gene transfer therapy infusion. A green circle  represents the acquisition of a developmental milestone (i.e., head holding, sitting for 30 seconds unassisted or standing for a few seconds with aid). A red circle represents the necessity of medical support (i.e., gastrostomy, NIV or spinal brace). An empty box represents the absence of developmental milestones or the need for medical support. The dashed boxes indicate that the data are not available at M24 (insufficient follow-up time). NIV stands for sleep NIV (i.e., <16 hr/day). The two patients who died are not represented. NIV: noninvasive ventilation, SMA: spinal muscular atrophy.

**Table 3:** Percentages of patients grouped by *SMN2* copy number reaching real-world endpoints (i.e., head holding, sitting 30 s unassisted, standing a few seconds with aid, or need for gastrostomy, NIV, or spinal brace) at M0 and M12 for all patients with 12 months of follow-up (2 deceased patients were excluded) [n = 27] (left column) and at M0, M12 and M24 for patients with 24 months of follow-up (2 deceased patients were excluded) [n = 17] (right)

SMN2 copies	12 months follow up		24 months follow up	
	2	3	2	3
	N = 20	N = 7	N = 13	N = 4
Head holding				
M0	45	86	31	75
M12	95	100	92	100
M24	–	–	100	100
Sitting 30s				
M0	0	0	0	0
M12	50	100	54	100
M24	–	–	85	100
Standing with aid				
M0	0	0	0	0
M12	10	14	8	0
M24	–	–	62	100
VNI				
M0	5	0	8	0
M12	30	0	23	0
M24	–	–	23	0
Gastrostomy				
M0	0	0	0	0
M12	10	0	8	0
M24	–	–	15	0
Spinal brace				
M0	0	0	0	0
M12	50	14	54	0
M24	–	–	85	100

All reported numbers above are percentages (%)

### Changes in motor scores and the TC/HC ratio after treatment with onasemnogene abeparvovec

**Fig. 2:**
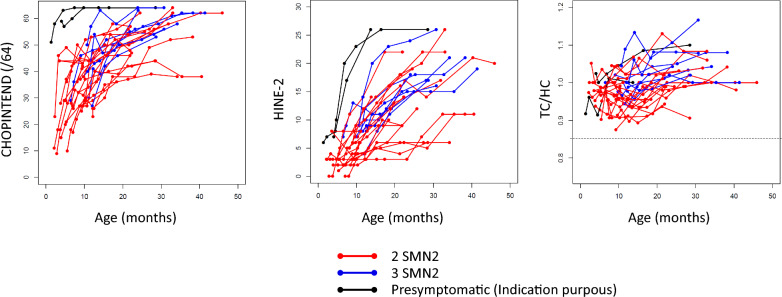
CHOPINTEND scores (left diagram), HINE-2 scores (middle) and TC/HC ratio (right) as a function of age for the 27 SMA patients (2 deceased patients excluded). TC: thoracic circumference, HC: head circumference. Red lines represent SMA patients with two copies of *SMN2,* and blue lines represent SMA patients with three copies of *SMN2*. For comparative purposes, the CHOPINTEND score, HINE-2 score and TC/HC ratio of the two presymptomatic patients with more than 12 months of follow-up in June 2022 are shown as black lines in the figure

**Fig. 3:**
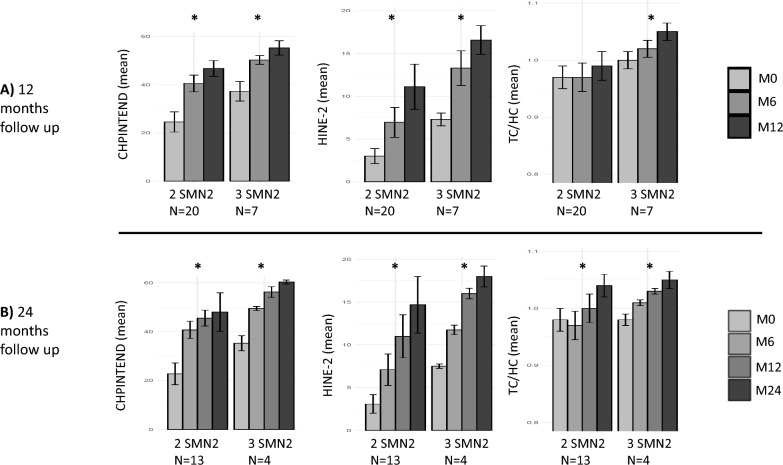
Mean CHOPINTEND scores (left diagram), HINE-2 scores (middle) and TC/HC ratio (right) **A** at M0, M6 and M12 for all patients with 12 months of follow-up [n = 27] (2 deceased patients excluded) and **B** at M0, M6, M12 and M24 for patients with 24 months of follow-up [n = 17] (2 deceased patients excluded). TC: thoracic circumference, HC: head circumference. * indicates a significant difference (*i.e.,* p value < 0.05 paired Student’s t test) between baseline and M12 (top 3 diagrams) or between baseline and M24 (bottom 3 diagrams)

Motor function improved in all patients, as shown in Fig. 2a (CHOPINTEND score) and Fig. 2b (HINE-2 score). The CHOPINTEND and HINE-2 scores were significantly different between M0 and M24 for patients with two and three *SMN2* copies, respectively (Fig. 3b). Between M12 and M24, little progress was observed on the CHOPINTEND scale, while HINE-2 continued to increase (Fig. 3b). This might have been due to saturation of the CHOPINTEND scale.

The TC/HC ratio increased in all patients (Fig. 2c). The lower limit of 0.85 (correlated with worse respiratory impairment [25, 26]) was never observed. The increase in the TC/HC ratio between M0 and M12 was not significant for patients with two *SMN2* copies, whereas it was significant for patients with three copies, strongly suggesting that the increase in thoraco-pulmonary growth was slower in patients with two *SMN2* copies.

### Adverse events after onasemnogene abeparvovec

Among the 29 patients treated with onasemnogene abeparvovec, one patient presented a generalized TMA on day 7 after GT (previously reported [16]). Intensive care was carried out, with hemofiltration and three eculizimab infusions. Despite this treatment, the patient died 6 weeks after the first symptoms. Another patient (SMA1a) developed severe respiratory failure 3 months after GT, requiring intensive care and permanent invasive ventilatory support without any improvement in the four following week. A withdrawal procedure was carried out after ethical committee discussions and parental consent.

Early biological follow-up revealed transaminase increases in all patients [ASAT from a mean of 42.5 IU at M0 to 74.6 IU at M2 (range 26–210) and ALAT from a mean of 29.3 IU at M0 to 69.8 IU at M2 (range 17–311 UI)] without hepatic failure, followed by complete normalization at M3 for 24/27 patients. The troponin I value increased in all patients, with a median value of 10 ng/l (range 10–55 ng/l) at M0 and a median value of 31 ng/l (range 31–75 ng/l) at M2 until normalization at M6. None of the patients had ECG or cardiac echography abnormalities.

Forty-seven percent of the patients with 24 months of follow-up had pneumopathies (range: 0 − 3 pneumopathies within 24 months of follow-up), and 26% were hospitalized at least once for respiratory infections (range: 0 − 1 hospitalization per year).

## Discussion

### Real-world outcomes

We report the real-world clinical outcomes of all SMA1 infants in France treated by GT as the first-line therapy between June 2019 and June 2022; all had had more than 12 months of follow-up. The GT indication was decided after thorough discussion by the FILNEMUS national expert committee. Patients with significant respiratory and/or bulbar impairment at diagnosis were oriented toward palliative care. This cohort of 29 infants, who were followed for at least 1 year and at most 2 years, allowed us to obtain real-world data from a homogeneous cohort of SMA1 patients treated only with GT, whereas previous reports were more heterogeneous and included patients initially or later treated with *SMN2* modifiers (nusinersen, risdiplam).

Concerning the 29 patients included in the follow-up study, motor function test (CHOPINTEND and HINE-2) results improved in all patients, in line with the findings of previous studies. Concerning key motor milestones, the outcome was also favorable, and at 2 years post-GT all 17 patients had acquired head support, almost 90% (15/17) were able to sit unassisted, and 12/17 were able to stand for a few seconds with aid.

The reported impact of these novel therapies on pulmonary well-being and the improving trajectory of pulmonary and bulbar morbidity, compared to the natural history of SMA, is particularly noteworthy in our series [8, 27]. The TC/HC ratio revealed favorable thoraco-pulmonary growth, with only three patients needing ventilation after 2 years of follow-up. This finding is strikingly different from our previous experience in the natural history of SMA1, during which a decreased ratio (TC/HC < 0.85) was observed and constituted a pejorative prognostic factor (Fig. 2) [25, 26]. These results suggest the fairly good health of the respiratory and bulbar muscles. In contrast, the results of several published studies evaluating nusinersen were less favorable [28–30]. However, it should be stressed that this cohort was probably less severe than that reported in previous nusinersen studies (no respiratory or bulbar dysfunction and a large proportion of SMA type 1c). Interestingly, our data suggest that the rate of thoraco-pulmonary growth was slower in patients with two *SMN2* copies than in those with three copies.

Despite the striking global motor improvements, spinal deformities were not prevented by GT, and almost all the patients required a spinal brace by the second year of follow-up. This underlines the observation that, although GT drastically improves motor outcome, patients still suffer from physically significant complications and disability. The brace indication is based on the clinical occurrence of scoliosis.

Our study did not allow proper comparisons of functional courses between patients with two or three *SMN2* copies since the severity of disease differed between these two groups at baseline. Nevertheless, our results support previous observations suggesting that SMA1 children with three *SMN2* mutations have less severe phenotypes and better outcomes.

### Clinical scores

Our data underline the importance of a multimodal motor score evaluation. In fact, between M12 and M24, little progress was observed on the CHOPINTEND scale, whereas more progress was observed on the HINE-2 scale. These differences might be because certain items of the CHOPINTEND are never reached by SMA patients, even after treatment (*e.g*., the item requiring strong posterior axial muscles of the neck to raise the head while facing the ground). Additionally, the CHOPINTEND does not test whether motor acquisition appears after 6 months of age. Our data suggest that motor development continues to progress between M12 and M24 because the HINE-2 score improved significantly and the proportion of patients sitting without support and standing increased from M12 to M24. Our criterion to add a second treatment line after GT was a 12-month stagnation period in motor scores. Due to the continuous increase in HINE-2 score in the patients of our cohort, however, no second line was initiated during the study period.

### National committee decision process and palliative care

Palliative care was proposed for 41 SMA1 patients, most of whom had SMA1a (34 SMA1a/41 SMA1) based on the clinical impression of a poor prognosis in patients with initial severe bulbar signs and respiratory distress in a no-return neurological disease. These patients were treated exclusively by supportive care and died in most cases over the next 6 months, as previously reported [26]. In all the cases, the decision not to administer GT to patients with respiratory insufficiency was made in accordance with the parents and the local legislation and for the main benefit of the child [24].

The age of treatment initiation in our GT cohort was delayed compared to that in the clinical trial [13] because our treatment criteria tended to include SMA type 1b and particularly 1c patients, who were consequently older at diagnosis and treatment. Motor development and the need for clinical support (*i.e.,* brace, NIV or gastrostomy) are in line with the findings of other studies reporting GT outcomes in SMA patients with similar disease severities [8].

Our strategy of systematic discussion with an online national expert committee did not delay treatment and enabled us to propose GT infusion within a short delay (mean delay: 15 days) after the clinical diagnosis was made. The systematic discussions with this online committee ensured consistent care of SMA1 patients all over the country, without disparities. As the committee is online, it easily brings together many experts for discussion and was not impacted by the SARS-CoV-2 pandemic, which occurred during the period of this study. Urgent meetings were sometimes organized, which shows the good reactivity of this national strategy. SMA1 therapy is an emergency due to the rapid disease progression and loss of function, which may occur within a few days, particularly in 1a and 1b infants.

In our study, we chose to analyze naive patients who had not received combined therapy for the first 12 months in order to evaluate the benefits of GT alone. In the literature, a number of patients treated with GT have been reported, most of whom were treated with nusinersen [7, 31]. All patients improved after GT, but it is difficult to assess the respective impact of each treatment and the benefits of their association in the combined therapies. The economic aspects and treatment costs also need to be considered [31, 32]. In our cohort, no second treatment was initiated because of continuous motor improvement.

### Severe adverse events

Early diagnosis of severe adverse events such as TMA is mandatory. This relies on close biological monitoring during the first 2 weeks, particularly regarding proteinuria, thrombocytosis, and elevated LDH and transaminases, so that therapies, such as a first-line increase in steroids and second-line eculizimab, can be started rapidly [16, 19].

### Prognostic factors

The presymptomatic children (both diagnosed because of a previous familial case) had normal motor development and a normal age at which they were able to walk. Early treatment and good initial motor scores were related to better outcomes according to our data, as previously reported [8]. A board of European experts has proposed age at treatment administration, respiratory impairment, motor level evaluated with CHOPINTEND, and *SMN2* copy number as prognostic factors. Our group recently underlined the prognostic importance of the median CMAP on motor function at M6 [33].

### Perspectives

The strength of our work is the presentation of real-world data concerning the efficacy of GT in a relatively large set of treatment-naive SMA1 patients from different regions of France through a national strategy. In the SMA population, the problems of a low number of patients in the cohorts, clinical heterogeneity, and treatment multiplicity will always persist. In this context, conventional randomized control trials are unlikely to be feasible. The French SMA registry has been systematically collecting data since September 2016. Additional long-term data analysis with statistical group homogenization will help clarify initial prognostic factors that impact functional outcomes. Finally, GT seems particularly effective for presymptomatic patients [14]. A national neonatal screening project is planned for the coming year and is still active in two pilot regions in France.

## Data Availability

In order to protect patients’ privacy under the French regulation (*CNIL*—*Commission nationale de l'informatique et des libertés*), the dataset supporting the conclusions of this research is only available upon reasonable request to the corresponding author (RB). No direct link to the dataset can be provided.

## Abbreviations

GT: Gene transfer therapy
NIV: Noninvasive ventilation
TC: Thoracic circumference
HC: Head circumference
PT/PC: Cranial circumference
FILNEMUS: French Rare Health Care for Neuromuscular Diseases Network
SMA: Spinal muscular atrophy
